# Prolonged use of noninvasive positive pressure ventilation after extubation among patients in the intensive care unit following cardiac surgery: The predictors and its impact on patient outcome

**DOI:** 10.1038/s41598-019-45881-x

**Published:** 2019-07-02

**Authors:** Pei-Ku Chen, Chun-Che Shih, Fang-Chi Lin, Diahn-Warng Perng, Kun-Ta Chou, Yu Ru Kou, Hsin-Kuo Ko

**Affiliations:** 1 Department of Chest Medicine, Taipei Veterans General Hospital, Taipei, Taiwan Republic of China; 2 School of Medicine, National Yang-Ming University, Taipei, Taiwan Republic of China; 3 Department of Cardiovascular Surgery, Taipei Veterans General Hospital, Taipei, Taiwan Republic of China; 4 Institute of Physiology, School of Medicine, National Yang-Ming University, Taipei, Taiwan Republic of China

**Keywords:** Risk factors, Medical research

## Abstract

This retrospective, observational cohort study aimed to determine the independent risk factors and impact of prolonged non-invasive positive pressure ventilation (NIPPV) after extubation among patients in the intensive care unit following cardiac surgery. Patients who received prophylactic NIPPV after extubation were categorized into prolonged (NIPPV duration >3 days, n = 83) and non-prolonged groups (NIPPV duration ≤3 days, n = 105). The perioperative characteristics and hospital outcomes were recorded. The multivariate analyses identified the preoperative residual volume/total lung capacity (RV/TLC) ratio (adjusted odds ratio [AOR]: 1.10; 95% CI:1.01–1.19, *p* = 0.022) and postoperative acute kidney injury (AKI) with Kidney Disease Improving Global Outcomes (KDIGO) stage 2–3, 48 h after surgery (AOR: 3.87; 95% CI:1.21–12.37, *p* = 0.023) as independent predictors of prolonged NIPPV. Patients with both RV/TLC ratio > 46.5% and KDIGO stage 2–3 showed a highly increased risk of prolonged NIPPV (HR 27.17, *p* = 0.010), which was in turn associated with higher risk of postoperative complications and prolonged ICU and hospital stays. Preoperative RV/TLC ratio and postoperative AKI could identify patients at higher risk for prolonged NIPPV associated with poor outcomes. These findings may allow early recognition of patients who are at a higher risk for prolonged NIPPV, and help refine the perioperative management and critical care.

## Introduction

Noninvasive positive pressure ventilation (NIPPV) has primarily been applied in patients with acute exacerbation of chronic obstructive pulmonary disease, worsening asthma and exacerbation, cardiogenic pulmonary edema, and hypoxemic acute respiratory failure (ARF)^1–3^. In the postoperative period, short-term use of NIPPV has been demonstrated to be beneficial for preventing (prophylactic use)^4–9^ and treating (therapeutic use) ARF after extubation^3,8–15^. Notably, patients undergoing cardiac surgery often encounter complications of significant pulmonary dysfunction postoperatively^13^. General anesthesia, cardiopulmonary bypass, and pain contribute to postoperative atelectasis after open heart surgery. Prophylactic use of NIPPV after extubation helps alleviate pulmonary function impairment and reduces the risk of postoperative pulmonary complications in these patients^5,8,9,14–16^. Several studies have focused on evaluating the safety and efficacy of prophylactic NIPPV in patients undergoing cardiac surgery^4,5^. Prolonged NIPPV after the intended prophylactic use in patients undergoing cardiac surgery seems to be an important issue in perioperative management and critical care. However, there is a paucity of data on the impact of and risk factors for prolonged NIPPV in this patient population.

There is a relatively frequent incidence of complications (15–30%) in patients undergoing cardiac surgery, and postoperative complications have been proven to be associated with increased resource utilization, morbidity (e.g., prolonged stay in intensive care unit (ICU) and prolonged hospitalization), and mortality^17–19^. Morbidity, mortality, and the development of postoperative complications are determined by several perioperative factors in patients undergoing cardiac surgery^17,18^. Although prolonged NIPPV after cardiac surgery could be a result of postoperative complications associated with cardiac and pulmonary dysfunctions^19^, prolonged NIPPV also potentially contributes to respiratory complications (e.g., pneumonia and respiratory muscle weakness) and subsequently worsens patient outcomes^19,20^. There is no study examining the relationship between prolonged NIPPV and patient outcomes including postoperative complications, morbidity, and mortality.

This study aimed to investigate the independent risk factors for prolonged NIPPV after extubation among patients in the ICU following cardiac surgery and determine the impact of prolonged NIPPV on patient outcomes.

## Results

### Characteristics of the study patients

**Figure 1:**
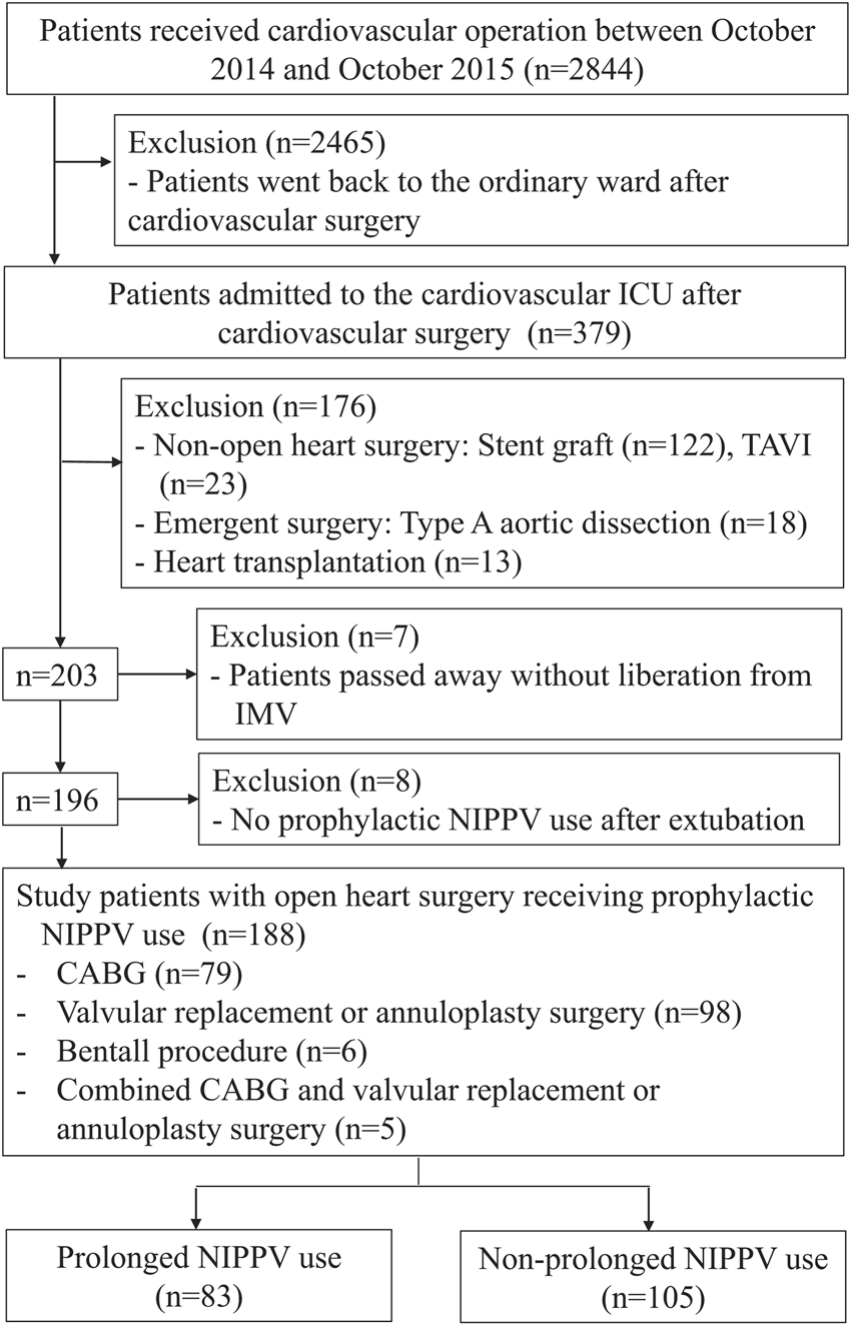
Study flowchart.

A total of 2844 patients underwent cardiovascular surgery during the study period, of which 379 patients admitted to the cardiovascular ICU after surgery were screened. The 86.7% of the patients undergoing cardiac surgery went back to the ordinary ward, and 13.3% (379/2844) patients were admitted to the ICU following cardiovascular surgery. After excluding 145 patients who underwent non-open-heart surgery (122 patients: stent graft and 23 patients: transcatheter aortic valve implantation [TAVI]), 18 who underwent Type A aortic dissection, 13 who underwent heart transplantation, 7 who passed away without liberation from invasive mechanical ventilation (IMV), and 8 who did not require prophylactic NIPPV after extubation, 188 patients were enrolled in this study (Fig. 1). All study participants were >18-years old and underwent open heart surgery, including coronary artery bypass surgery (CABG, n = 79), valvular replacement or annuloplasty surgery (n = 98), and others (n = 11). The median age of the study patients (126 men, 62 women) was 63 (52–72) years, and the mean APACHE II score on ICU admission was 28.2 (±4.7). The median duration of NIPPV was 3 (1–8) days, and 44.1% (83/188) study patients received NIPPV for more than 3 days. Among the study patients, 50.5% (95/188) developed acute kidney injury (AKI) within 48 h after cardiac surgery (Kidney Disease Improving Global Outcomes; KDIGO Stages 1, 2, and 3; n = 60, 15, 20, respectively), and 29.8% (56/188) developed postoperative complications in a median of 10 days (3–23) after cardiac surgery. The study patients were categorized into prolonged NIPPV (n = 83) and non-prolonged NIPPV (n = 105) groups.

### Risk factors for prolonged NIPPV after cardiac surgery

The demographic characteristics of the study patients with and without prolonged NIPPV are shown in Table 1. Patients with prolonged NIPPV after cardiac surgery had significantly lower values of forced expiratory volume in 1 s (FEV1) % predicted (70.0% vs. 84.7%, *p* < 0.001), forced vital capacity (FVC) % predicted (67.0% vs. 81.3%, *p* < 0.001), total lung capacity (TLC) % predicted (84.9% vs. 91.5%, *p* = 0.010), inspiratory capacity (IC) % predicted (69.6 vs. 83.4%, *p* < 0.001), and diffusing capacity for carbon monoxide (DL_CO_) % predicted (51.6% vs. 60.4%, *p* = 0.005), higher residual volume (RV)/TLC ratio (49.2% vs. 41.8%, *p* < 0.001), lower preoperative left ventricular ejection fraction (54% vs. 58%, *p* = 0.017), higher baseline creatinine level (1.19 vs. 1.00 mg/dL, *p* = 0.008), lower hemoglobin level (11.8 vs. 12.8 g/dL, *p* = 0.025), and lower serum albumin level (3.5 vs. 3.8 mg/dL, *p* = 0.002) than those without prolonged NIPPV.

**Table 1:** Baseline demographic characteristics of the study patients.

Variable	All (n = 188)	Prolonged NIPPV (n = 83)	Non-prolonged NIPPV (n = 105)	*P*
Age (y)	63 (52–72)	66 (51–73)	60 (53–69)	0.109
Male	126 (67.0)	54 (65.1)	72 (68.6)	0.611
BMI (kg/m^2^)	24.5 (22.3–27.2)	24.0 (22.3–28.8)	24.7 (22.4–27.5)	0.522
Smoking	64 (34.2)	25 (30.5)	39 (37.1)	0.341
**Comorbidities**				
Vascular disease	37 (19.7)	19 (22.9)	18 (17.1)	0.325
Pulmonary disease	13 (6.9)	4 (4.8)	9 (8.6)	0.393
Cancer	14 (7.4)	6 (7.2)	8 (7.6)	0.919
hypertension	109 (57.9)	44 (53.0)	65 (61.9)	0.220
CHF	37 (19.7)	16 (19.3)	21 (20.0)	0.901
Pulmonary HTN	15 (8.0)	4 (4.8)	11 (10.5)	0.184
Diabetes mellitus	66 (35.1)	32 (38.6)	34 (32.4)	0.379
CKD	28 (14.9)	17 (20.5)	11 (10.5)	0.065
**Spirometry** (**n = 141**)				
FEV1 (% pred)	79.3 ± 21.7	70.0 ± 22.5	84.7 ± 19.5	<0.001
FVC (% pred)	76.1 ± 20.1	67.0 ± 20.8	81.3 ± 17.8	<0.001
FEV1/FVC (%)	79.5 ± 8.7	79.7 ± 8.4	79.5 ± 9.0	0.890
**Lung volume** (**n = 135**)				
RV (% pred)	111.2 ± 20.6	115.5 ± 25.0	108.8 ± 17.4	0.073
TLC (% pred)	89.2 ± 14.4	84.9 ± 15.7	91.5 ± 13.1	0.010
RV/TLC (%)	44.5 ± 9.6	49.2 ± 10.3	41.8 ± 8.2	<0.001
IC (% pred)	78.5 ± 20.2	69.6 ± 20	83.4 ± 18.6	<0.001
DL_CO_ (%pred) (n = 114)	57.3 ± 16.3	51.6 ± 15.5	60.4 ± 16.0	0.005
DL_CO_/VA(%pred)	74.6 ± 16.9	73.3 ± 15.9	75.3 ± 17.5	0.538
**Heart echo** (**n = 142**)				
LVEF	56 (50–61)	54 (48–59)	58 (53–61)	0.017
RVSP	39 (29–56)	43 (33–58)	37 (28–55)	0.105
Diastolic HF	71 (48.3)	30 (52.6)	41 (45.6)	0.403
**Laboratory data**				
Hemoglobin (g/dl)	12.5 (10.8–13.7)	11.8 (10.2–13.6)	12.8 (11.3–13.8)	0.025
Creatinine (mg/dl)	1.04 (0.83–1.41)	1.19 (0.85–1.89)	1.00 (0.82–1.23)	0.008
Albumin (g/dl)	3.7 (3.2–4.0)	3.5 (3.1–3.9)	3.8 (3.4–4.1)	0.002

Data are reported as mean ± SD or median (interquartile range) or number (%).

CHF = congestive heart failure, HTN = hypertension, CKD = chronic kidney disease, FEV1 = forced expiratory volume in one second, FVC = forced vital capacity, RV = residual volume, TLC = total lung capacity, IC = inspiratory capacity, DL_CO_ = diffusing capacity of the lung for carbon monoxide, VA = vital capacity, LVEF = left ventricular ejection fraction, RVSP = right ventricular systolic pressure.

The procedural and postoperative characteristics of the study patients are shown in Table 2. Patients with prolonged NIPPV use after cardiac surgery had a significantly higher APACHE II scores on ICU admission (29.5 vs. 27.3, *p* = 0.001), longer duration of IMV (2 days vs. 1 day, *p* < 0.001), higher percentage of impaired renal functionl (KDIGO Stage 2–3) within 48 h after surgery (25.6% vs. 13.3%, *p* = 0.033), and higher operative procedure-related complications (20.5 vs. 9.5%, *p* = 0.033).

**Table 2:** Procedural and postoperative characteristics of the study patients.

Variable	All (n = 188)	Prolonged NIPPV (n = 83)	Non-prolonged NIPPV (n = 105)	*p*
APACHE II on ICU admission	28.2 ± 4.7	29.5 ± 5.1	27.3 ± 4.2	0.001
Combined surgery	51 (27.1)	25 (30.1)	26 (24.8)	0.412
Operation time (mins)	310 (240–385)	300 (230–390)	325 (240–385)	0.612
Bypass times (mins)	144 (110–223)	148 (111–230)	141 (110–214)	0.768
Aortic clamp times (mins)	95 (70–146)	84 (61–156)	99 (71–138)	0.524
**ABG after surgery**				
PaO2/FiO2	275 ± 131	283 ± 141	268 ± 123	0.419
pH	7.32 ± 0.08	7.33 ± 0.09	7.31 ± 0.07	0.178
PaCO2	44.2 ± 23.0	42.6 ± 8.5	45.4 ± 29.9	0.398
HCO3	22.3 ± 3.4	22.8 ± 3.7	21.9 ± 3.2	0.090
**AKI after cardiac surgery**				
KDIGO stage 1	60 (31.3)	26 (31.7)	34 (32.4)	0.516
KDIGO stage 2–3	35 (18.7)	21 (25.6)	14 (13.3)	0.033
Operative procedure-related complications	27 (14.4)	17 (20.5)	10 (9.5)	0.033
RSBI (n = 141)	61.3 ± 30.1	65.3 ± 31.7	57.6 ± 28.3	0.129
Duration of IMV (day)	1 (0–5)	2 (1–12)	1 (0–2)	<0.001
**ABG after extubation**				
pH	7.42 ± 0.06	7.42 ± 0.06	7.42 ± 0.06	0.888
PaCO2	38.13 ± 6.1	38.65 ± 6.4	37.61 ± 5.9	0.278
HCO3	24.66 ± 4.2	24.97 ± 4.3	24.3 ± 4.0	0.330
Postoperative complications	56 (29.8)	39 (47.0)	17 (16.2)	<0.001
Pneumonia	31 (16.5)	23 (27.7)	8 (7.6)	<0.001
Arrhythmia	8 (4.3)	7 (8.4)	1 (1.0)	0.023
GI bleeding	5 (2.7)	5 (6.0)	0 (0)	0.016
stroke	13 (6.9)	11 (13.3)	2 (1.9)	0.003
Ischemic bowel	4 (2.1)	2 (2.4)	2 (1.9)	1
Reintubation	17 (9.0)	11 (7.5)	6 (5.7)	0.073
ICU stay (day)	4 (2–10)	8 (4–21)	3 (2–5)	<0.001
Hospital stay (day)	21 (15–38)	32 (21–53)	17 (12–23)	<0.001
Hospital mortality	11 (5.9)	7 (8.4)	4 (3.8)	0.219

Data are reported as mean ± SD or median (interquartile range) or number (%).

APACHE II = Acute physiology and chronic health evaluation II, ICU = intensive care medicine, ABG = arterial blood gas, KDIGO = Kidney disease- Improving global outcomes, AKI = Acute kidney injury, RSBI = Rapid shallow breathing index, IMV = Invasive mechanical ventilation, GI bleeding = Gastrointestinal bleeding.

### Independent factors for predicting prolonged NIPPV after cardiac surgery

The finally adjusted multivariate logistic regression model showed that the independent factors predictive for prolonged NIPPV were RV/TLC ratio (AOR 1.10, 95%; confidence interval, CI: 1.01 to 1.19, *p* = 0.022) and AKI (KDIGO Stage 2–3; AOR 3.87, 95% CI: 1.21 to 12.37, *p* = 0.023) (Table 3).

**Table 3:** Univariate and multivariate logistic regression analysis of risk factors for the prolonged noninvasive positive pressure ventilation in the study patients (n = 188).

Variable	Univariate		Simplified model using backward elimination method^a^		Fully adjusted model adding important confounders^b^	
	OR (95%CI)	*p*	AOR (95% CI)	*p*	AOR (95%CI)	*p*
Age	1.02 (1.00–1.04)	0.134	_^*a*^		1.00 (0.95–1.04)	0.815
Sex	0.85 (0.46–1.57)	0.611	_^*a*^		0.86 (0.28–2.59)	0.784
BMI	0.99 (0.94–1.05)	0.801	0.88 (0.78–0.99)	0.039	0.89 (0.78–1.01)	0.070
APACHE II	1.11 (1.04–1.19)	0.002	_^*a*^		1.02 (0.88–1.19)	0.775
Duration of IMV	1.14 (1.07–1.21)	<0.001	1.10 (1.02–1.19)	0.019	1.08 (0.99–1.18)	0.072
FEV1%pred	0.97 (0.95–0.98)	<0.001	_^*a*^		-^*b*^	
FVC %pred	0.96 (0.94–0.98)	<0.001	_^*a*^		-^*b*^	
TLC %pred	0.97 (0.94–0.99)	0.012	_^*a*^			
RV/TLC (%)	1.09 (1.05–1.14)	<0.001	1.08 (1.04–1.13)	<0.001	1.10 (1.01–1.19)	0.022
IC %pred	0.96 (0.94–0.98)	<0.001	_^*a*^			
DL_CO_ %pred	0.97 (0.94–0.99)	0.007	_^*a*^		0.99 (0.95–1.03)	0.575
Hemoglobin	0.85 (0.74–0.98)	0.024	_^*a*^		1.00 (0.75–1.34)	0.993
KDIGO stage 2–3	2.24 (1.06–4.74)	0.035	4.19 (1.42–12.39)	0.010	3.87 (1.21–12.37)	0.023
Albumin	0.40 (0.22–0.73)	0.002	2.49 (0.85–7.27)	0.096	2.83 (0.89–9.02)	0.079
Operative procedure-related complication	2.45 (1.05–5.68)	0.037	_^*a*^		1.62 (0.43–6.04)	0.475
Creatinine	1.12 (0.99–1.27)	0.078				
LVEF	0.97 (0.93–1.00)	0.074				
RVSP	1.01 (1.00–1.03)	0.143				

Data are reported as mean ± SD or median (interquartile range) or number (%).

BMI = body mass index, APACHE = acute Physiology and Chronic Health Evaluation, IMV = invasive mechanical ventilation, FEV1 = forced expiratory volume in one second, FVC = forced vital capacity, TLC = total lung capacity, RV = residual volume, IC = inspiratory capacity, DLco = diffusing capacity, KDIGO = Kidney Disease Improving Global Outcomes, LVEF = left ventricular ejection fraction, RVSP = right ventricular systolic pressure.

^*a*^Variables entered into multivariate logistic regression analysis with backward elimination method did not retain in the final model.

^*b*^The continuous variable, FEV1%pred, is highly correlated with FVC %pred (Pearson correlation coefficient 0.881, p < 0.001); thus, they must be excluded from each other before entry in the fully adjusted model. RV/TLC is also correlated with FEV1%pred (Pearson correlation coefficient −0.671, p < 0.001) and FVC %pred (Pearson correlation coefficient −0.761, p < 0.001). Both of the two variables (FEV1% pre and FVC % pred) were removed in the backward elimination procedure. Therefore, we removed them in the fully adjusted model.

### Synergistic impact of RV/TLC ratio and KDIGO stage on prolonged NIPPV

**Figure 2:**
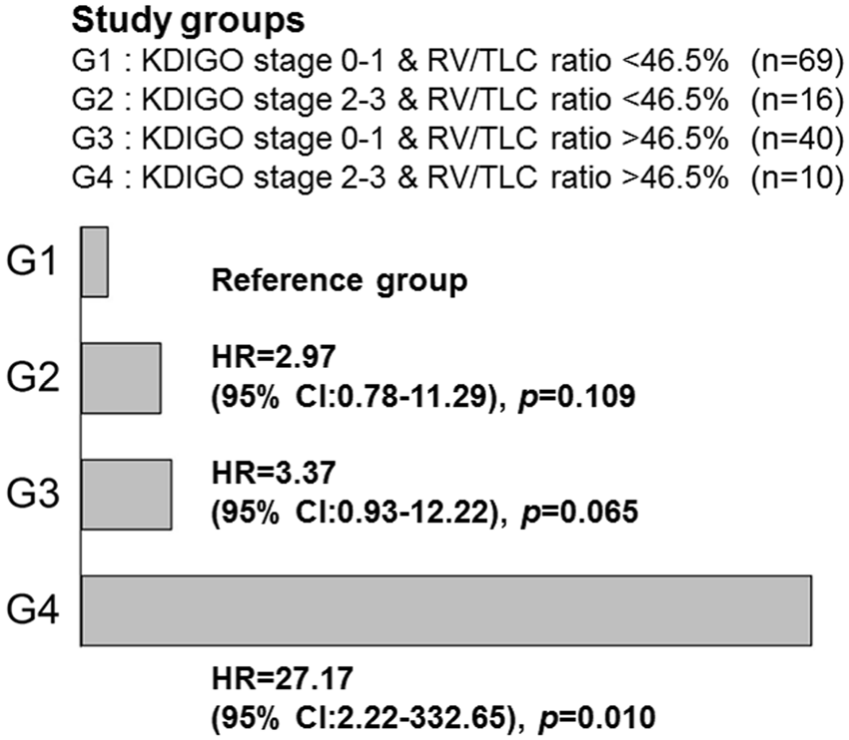
The synergistic impact of residual volume to total lung capacity (RV/TLC) ratio and KDIGO stage of AKI on the risk of prolonged NIPPV. G1 group, KDIGO Stage 0–1 and RV/TLC ratio < 46.5% (n = 69); G2 group, KDIGO Stage 2–3 and RV/TLC ratio < 46.5% (n = 16); G3 group, KDIGO Stage 0–1 and RV/TLC ratio > 46.5% (n = 40); G4 group, KDIGO Stage 2–3 and RV/TLC ratio > 46.5% (n = 10). The corresponding Cox hazard ratios (HRs) with 95% confidence intervals (95% CIs) for prolonged NIPPV use after cardiac surgery are shown.

The ROC curve analysis was estimated for RV/TLC ratio, and a cut-off level of 46.5% was chosen while differentiating between prolonged and non-prolonged NIPPV after cardiac surgery (area under curve = 0.72, sensitivity = 60.4%, specificity = 75.9%, *p* < 0.001). The synergistic effect of RV/TLC ratio and AKI on prolonged NIPPV was analyzed by Cox regression analysis. Patients were divided into four groups as below: G1: KDIGO Stage 0–1 and RV/TLC ratio < 46.5% comprised the reference group, G2: KDIGO Stage 2–3 and RV/TLC ratio < 46.5%, G3: KDIGO Stage 0–1 and RV/TLC ratio > 46.5%, and G4: KDIGO Stage 2–3 and RV/TLC ratio > 46.5%. The HR dramatically increased and reached statistical significance for G4 patients compared with that for the G1 patients (HR 27.17, 95% CI: 2.22 to 332.65, *p* = 0.010) (Fig. 2). These two independent factors were found to have synergistic effects in that the risk of prolonged NIPPV was exponentially increased in patients with both risk factors.

### The impact of prolonged NIPPV use on patient outcomes

A higher proportion of patients with prolonged NIPPV had postoperative complications (47.0% vs. 16.2%, *p* < 0.001), postoperative pneumonia (27.7% vs. 7.6%, *p* < 0.001), arrhythmia (8.4 vs. 1.0%, *p* = 0.023), gastrointestinal bleeding (6.0 vs. 0%, *p* = 0.016) stroke (13.3 vs. 1.9%, *p* = 0.003), and longer duration of ICU stay (8 vs. 3 days, *p* < 0.001) and hospital stay (32 vs. 17 days, *p* < 0.001) than those without prolonged NIPPV (Table 2).

**Figure 3:**
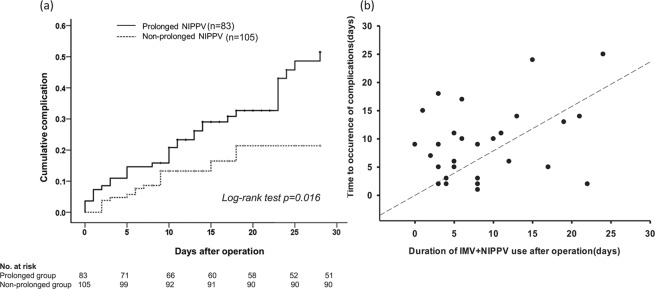
(**a**). Kaplan–Meier curves of the probability of postoperative complication after operation depending on prolonged and non-prolonged NIPPV after cardiac surgery, (**b**). The distribution of the duration of invasive mechanical ventilation plus noninvasive positive pressure ventilation (IMV + NIPPV) and the day of development of a complication after cardiac surgery. Within postoperative 28 days, more than half of the patients (17/31) developed complications after discontinuation of ventilation support.

The relationship between prolonged NIPPV and patient outcomes by multivariate logistic regression and the odd ratios (ORs) and AORs for patient outcomes are shown in Table 4. Compared with non-prolonged NIPPV, prolonged NIPPV was independently associated with an increased risk of postoperative complications (AOR 4.46, 95% CI: 1.56 to 12.79, *p* = 0.005) and prolonged ICU stays of >7 days (AOR 18.17, 95% CI: 3.10 to 106.45, *p* = 0.001) and hospital stays of >21 days (AOR 4.44, 95% CI: 1.12 to 17.57, *p* = 0.034). The relationship between prolonged NIPPV and the probability of postoperative complications is shown in Fig. 3a. The prolonged NIPPV group had more postoperative complications (long-rank test *p* = 0.016). The distribution of ventilator days (IMV and NIPPV) and day of development of complications is shown in Fig. 3b. The finding indicated that prolonged NIPPV had a critical impact on sequential development of postoperative complications and morbidity (longer length of ICU and hospital stay), but not on mortality.

**Table 4:** Univariate and multivariate logistic regression analysis to evaluate the impact of prolonged noninvasive positive pressure ventilation use on patient outcomes^*a*^.

Patient outcomes	Univariate OR	*p*	Adjusted OR^*b*^	*p*
Postoperative complications	4.59 (2.34–9.01)	<0.001	4.46 (1.56–12.79)	0.005
Pneumonia	4.65 (1.95–11.06)	<0.001	2.69 (0.70–10.36)	0.151
Arrhythmia	9.58 (1.15–79.49)	0.036	1.22 (0.19–7.90)	0.838
Stroke	7.87 (1.69–36.57)	0.009	2.12 (0.25–17.71)	0.490
Ischemic bowel	1.27 (0.18–9.22)	0.812	1.10 (0.12–10.01)	0.930
Reintubation	2.52 (0.89–7.13)	0.081	1.58 (0.24–10.55)	0.635
ICU stay >7 days	5.30 (2.70–10.41)	<0.001	18.17 (3.10–106.45)	0.001
Hospital stay >21 days	5.29 (2.83–9.90)	<0.001	4.44 (1.12–17.57)	0.034
Hospital mortality	2.33 (0.66–8.23)	0.191	0.78 (0.15–4.10)	0.767

Data were reported as odds ratio (95% confidence interval). ^*a*^non-prolonged NIPPV group as reference. ^*b*^Variables significantly associated with patient outcomes on univariate analysis (*p* < 0.05) were included in the multivariate logistic regression analysis. NIPPV = noninvasive positive pressure ventilation, OR = odds ratio, ICU = intensive care unit.

## Discussion

Our study reported that the preoperative RV/TLC ratio and AKI of KDIGO Stage 2–3 within 48 h postoperatively were independent predictive factors for prolonged NIPPV after extubation among patients in the ICU following cardiac surgery. These two risk factors seem to act synergistically for prolonged NIPPV after cardiac surgery. Of note, prolonged NIPPV was independently associated with poor patient outcomes including an increased risk of postoperative complications, and prolonged ICU and hospital stays. We suggest that an RV/TLC ratio cut-off value of greater than 46.5% and AKI of KIDGO Stage 2–3 can be used by intensivists to preoperatively and postoperatively identify patients who are at a high risk of prolonged NIPPV after cardiac surgery. Our findings may allow early recognition of patients being at a higher risk for prolonged NIPPV and help refine the perioperative management and critical care.

In total, 40 to 90% of patients undergoing cardiac surgery have been reported to experience pulmonary complications^21^. Non-invasive ventilations are clinically applied to reduce the adverse events after cardiac surgery in these patients. Both continuous positive airway pressure (CPAP) and bilevel NIPPV, if applied after evaluation of surgical complications, are safe and can reduce re-intubation rates, morbidity, and mortality after cardiac surgery^8,22^. In randomized trials, the application of prophylactic CPAP or NIPPV compared to standard treatment with oxygen therapy and chest physiotherapy for a period of 1–12 h improved the gas exchange^5,16,21,23^ and functional capacity^9^, but there was no statistical difference in the spirometry^22^, or rate of atelectasis^22,24^. However, Zarbock *et al*. conducted a large randomized trial enrolling 468 patients, and showed that prophylactic CPAP compared to oxygen therapy significantly reduced the incidence of hypoxemia and pneumonia and the rate of reintubation, but not the lengths of stay in the ICU and hospital^16^. These findings indicate the benefit of prophylactic CPAP or NIPPV use in improving gas oxygen exchange after cardiac surgery. In an analysis of the application of therapeutic CPAP or NIPPV use for atelectasis or respiratory failure, Pasquina *et al*. enrolled 150 patients presenting a radiological atelectasis score >2 after cardiac surgery and randomized them to receive either CPAP or NIPPV^25^. As compared to CPAP, NIPPV led to a significantly higher reduction in the radiological atelectasis score, but there was no difference in arterial blood gas, the length of hospital stay, and the mortality rate between the two groups. De Morasis Coimbra *et al*. enrolled 57 patients with acute respiratory failure after cardiac surgery to receive either CPAP or NIPPV^26^. Their result did not show a statistical difference in gas exchange or reduction of intubation rate between the two groups. The data from these literatures implied that CPAP and NIPPV could be considered as prophylactic and therapeutic tools to improve gas exchange and functional capacity in post-cardiac surgery patients.

Static lung volume tests including the TLC, RV, and functional residual capacity are clinically used to make a diagnosis of restrictive lung diseases and to determine the presence of lung hyperinflation or air trapping^27^. In the current study, we identified that increased RV/TLC ratio predicted prolonged NIPPV after cardiac surgery. Theoretically, lung hyperinflation and/or air trapping in patients with lung function with increased RV/TLC ratio could increase respiratory workload and potentially contribute to prolonged NIPPV after cardiac surgery. Spirometry has been used preoperatively to detect obstructive lung disease^28^ and predict postoperative morbidities and mortality in patients undergoing cardiac surgery^29–31^. Preoperative FEV1/FVC ratio was not a sensitive factor for predicting prolonged NIPPV support after cardiac surgery in our study. The result could be explained by the patient characteristics. A higher proportion of patients in our study had coronary artery disease, valvular heart disease, and other heart diseases, commonly revealing restrictive lung disease in these patients; the degree of airway obstruction evaluated by forced spirometry in such patients is often underestimated^32^. This may be the explanation for RV/TLC ratio being a sensitive marker for simultaneously detecting lung diseases and predicting prolonged NIPPV after cardiac surgery. Our finding suggested that total lung volume test provided a more physiological baseline condition and it was an important factor for preoperative evaluation.

AKI has been identified as the independent risk factor for prolonged IMV in critically ill patients^33,34^. Several studies have reported that AKI occurs in approximately 25% of patients undergoing cardiac surgery^35–37^ and is a proven independent risk factor for operative and late mortality in this patient population^38–41^. Cardiac surgery-associated AKI (CS-AKI) affects major outcomes and is an important issue for clinicians^41^. In our study, 50.5% of study patients developed AKI (KDIGO Stage 1–3) within 48 h after surgery. The incidence of AKI in the current study was similar to that in Machado’s study (43%)^39^, but it was double that in most other previous studies^35–37^. Several factors alter renal perfusion during the operative procedure; for example, cardiopulmonary bypass, aortic cross-clamping, blood loss, and high volumes of exogenous blood product transfusion are implicated in the development of AKI and are potentially modifiable^42^. If these modifiable factors contributing to AKI following cardiac surgery can be controlled, it may prevent the development of AKI associated with fluid retention and overload, and thereby possibly reduce prolonged NIPPV after cardiac surgery.

Our study also reported that the independent factors of RV/TLC ratio and AKI exponentially increased the risk of prolonged NIPPV after cardiac surgery in patients with both risk factors. In critically ill elderly patients, Pan *et al*. report the synergistic impact of low serum albumin on ICU admission and high blood urea nitrogen during ICU stay on post-ICU mortality^43^. In acute traumatic spinal cord injury patients, Yu *et al*. report the synergistic impacts of AKI and high level of cervical spinal cord injury on the ventilator weaning outcomes^34^. These findings highlight the fact that multiple factors may have a synergistic effect on hospital and post-discharged outcomes in critically ill patients. Therefore, intensivists should evaluate these factors simultaneously in critically ill patients during and after surgery while they are receiving NIPPV and IMV during ICU stay.

In this study, we reported that patients undergoing cardiac surgery and requiring prolonged NIPPV had a significantly increased risk of postoperative complications, ICU stay >7 days and hospital stay >21 days. These findings indicated that the prophylactic use of NIPPV theoretically reduced the risk of postoperative surgical complications and the occurrence of ARF. However, patients who required prolonged NIPPV for durations over 72 h were at a significantly increased risk of postoperative complications and morbidity (longer ICU and hospital stays). Our study patients had a longer ICU and hospital stay than those among patients in recent studies^44–46^. Many factors, such as age, renal failure, and underlying disease influence the length of stay^47^. The variability in the baseline and perioperative characteristics may contribute to the differences in the length of stay. Compared with other cohorts^48,49^, a higher APACH II score at ICU admission may be the reason for the longer ICU stay in our study patients.

Our study had several limitations. First, this was a single-center retrospective cohort study. Bias and non-standardized procedures existed. Some data was missing and could not be recorded. For example, details regarding preoperative medications before patient admission can not be fully discovered. Thus, the effects of preoperative medications could not be evaluated in this study. Second, the duration of NIPPV was defined based on the need of NIPPV for at least 6 h every day, the duration of the prophylactic use of NIPPV after extubation may show some differences. Third, as there is no consensus on the definition of “prolonged” NIPPV duration, we calculated the median NIPPV duration among the study subjects and used it to define prolonged NIPPV. Fourth, we excluded more critically ill patients (ex: emergent surgery for Type A aortic dissection and heart transplantation), and the impact of prolonged NIPPV in such groups could not be identified in this study. Finally, this is a relatively small case series conducted in a short period. Almost all the subjects admitted in the ICU following open heart surgery were administered prophylactic NIPPV. Future validation and prospective methods for standardization of the clinical protocols are warranted.

## Conclusion

We reported the RV/TLC ratio and AKI of KDIGO Stage 2–3 as the independent factors to predict the requirement of prolonged NIPPV after cardiac surgery. Prolonged NIPPV in patients undergoing cardiac surgery was significantly associated with a higher risk of postoperative complications and prolonged ICU and hospital stays. Our findings may achieve early recognition of patients being at a higher risk for prolonged NIPPV, and then refine the perioperative management and critical care in patients undergoing cardiac surgery.

## Methods

### Design, patients, and setting

This observational retrospective study was conducted in the cardiovascular surgery ICU (a 16-bed ICU) of Taipei Veteran General Hospital (Taiwan), a 3000-bed tertiary medical center, and was approved by the Institutional Ethical Review Board of Taipei Veterans General Hospital (VGHTPE-IRB No. 2017-05-001AC). Informed consent was waived under the approval of our IRB according to the institutional guidelines for a retrospective observational study. Patients were extubated in the operating room (early extubation) or ventilated in the ICU (late extubation) depending on the clinical condition. Rapid shallow breathing index (frequent/tidal volume) before extubation was checked. In the late extubation group, patients received spontaneous breathing trials (SBTs) with low levels of pressure support (5–8 cm H_2_O). IMV was discontinued while SBTs passed. Patients who were admitted to the cardiovascular ICU following cardiovascular surgery between October 2014 and October 2015 were reviewed. To reduce the potential confounding factors, patients were excluded from the study if they 1) had undergone a less invasive surgery than an open heart surgery, such as stent graft surgery and TAVI, 2) had undergone an emergent surgery such as Type A aortic dissection without a complete preoperative survey, 3) had undergone heart transplantation because of a high degree of disease severity, or 4) did not receive prophylactic NIPPV after surgery. In addition to cardiac surgeons, two pulmonologists (Dr. Ko and Dr. Lin) participated in critical care and managed the invasive/noninvasive ventilator during the study period. Finally, patients who were admitted to the cardiovascular ICU following cardiac surgery and received prophylactic NIPPV after extubation during the study period were enrolled. The study patients were classified into prolonged and non-prolonged NIPPV groups.

### Measurement

The data including baseline characteristics, acute Physiology and Chronic Health Evaluation (APACHE) II score on ICU admission^50^, and perioperative profiles were extracted from medical charts and electronic medical records. During the postsurgical period, the duration of ventilator use and clinical events were recorded.

### Definitions

Prophylactic NIPPV after surgery was defined as noninvasive ventilator support with the application of bilevel positive airway pressure following extubation for at least 6 h on the first day of liberation from IMV^15,16^. Criteria of continuing NIPPV was based on the following clinical conditions: (1) severe dyspnea with accessory muscle use, (2) respiratory acidosis with pH less than 7.35 and/or partial pressure of carbon dioxide more than 45 mmHg, and (3) tachypnea with a respiratory rate more than 30 times/ min. The median duration of NIPPV in our study population was 3 (range, 1–8) days; therefore, prolonged NIPPV was defined as the requirement of NIPPV for at least 6 h every day for a period of more than 3 days. Postoperative laboratory data within 48 h after surgery were collected. AKI was defined according to the KDIGO guidelines^51^ and was categorized as follows: (1) Stage 1: serum creatinine level increased by 1.5–1.9 times the baseline value or serum Cr ≥ 0.3 mg/dL within 48 h; (2) Stage 2: serum creatinine level increased by 2.0–2.9 times the baseline value; and (3) Stage 3: serum creatinine level increased by more than 3 times the baseline value or to >4.0 mg/dL or initiation of renal replacement therapy. Arterial blood gas (ABG) was recorded as the first ABG data after surgery and after discontinuation of IMV in the ICU. Operative procedure-related complications were defined as events that occurred within 1 week after the operation including major bleeding, extravasation with cardiac tamponade, hemothorax, and sternal wound breakdown, among others. Postoperative complications were defined as the occurrence of pneumonia, arrhythmia, gastrointestinal bleeding, stroke, ischemic bowel disease, reintubation, and other critical events during ICU stay.

### Statistical analysis

The Kolmogorov–Smirnov test was used to check the distribution of continuous variables. Continuous variables were described as means (±standard deviation, SD) or medians (interquartile ranges, IQRs), as appropriate. Categorical variables were described as percentages. Student’s *t*-test was used for normally distributed variables. Mann–Whitney U test was used to compare continuous variables with a non-normal distribution. Chi-square or Fisher’s exact test was used to compare percentages. Univariate analysis was performed to analyze the clinical factors associated with prolonged NIPPV. Variables significantly associated with prolonged NIPPV (*p* < 0.05) on univariate analysis were included in multivariate logistic regression analysis. Backward elimination was employed to identify the significant predictors. Subsequently, a fully adjusted multivariate logistic/Cox regression analysis was conducted including the important confounding factors. We considered a two-tailed *p* value of <0.05 to indicate significance, ORs and hazard ratios (HRs) with 95% CIs were calculated. When a continuous variable was found to have significance on multivariate Cox regression analysis, a receiver operating characteristic (ROC) curve analysis was used to check the optimal cut-off point to create dichotomous variables. We analyzed the relationship between prolonged NIPPV and the occurrence of postoperative complications by Kaplan–Meier analysis and log–rank test. Statistical analysis was performed using SPSS version 20.0 (SPSS, Chicago, IL, USA).
